# Insuffisance rénale aigüe: présentation rare d’une maladie d’Addison

**DOI:** 10.11604/pamj.2016.24.233.9974

**Published:** 2016-07-13

**Authors:** Houda Salhi

**Affiliations:** 1 Division of endocrinology, Provincial hospital, Guercif 35100, Morroco

**Keywords:** Insuffisance rénale aiguë, maladie d´Addison, myélome multiples, Acute renal failure, Addison’s disease, multiple myeloma

## Abstract

La maladie d’Addison est une pathologie rare, qui se manifeste fréquemment par des signes cliniques non spécifiques. Ce qui peut causer un retard diagnostic et thérapeutique. Cette maladie peut se présenter comme un tableau d’insuffisance rénale aigue. Nous rapportons le cas d’un patient présentant une maladie d’Addison qui a été pris en charge initialement comme une insuffisance rénale aigue secondaire à un myélome multiple et dont le diagnostic a été redressé par la suite. Le patient s’est spectaculairement amélioré après mis en place de traitement par réhydratation par voie intraveineuse; hydrocortisone injectable.

## Abstract (en)

Addison’s disease is a rare condition. Its onset of symptoms most often is nonspecific contributing to a diagnostic and therapeutic delay. Acute renal failure can be the first manifestation of this disease. We report the case of a patient with Addison’s disease who was initially treated for acute renal failure due to multiple myeloma and whose diagnosis was adjusted thereafter. Patient’s condition dramatically improved after treatment with intravenous rehydration; injectable hydrocortisone.

## Introduction

L’insuffisance surrénalienne primaire ou « maladie d’Addison » décrite pour la première fois par Thomas Addison [1] se défini par un déficit de sécrétion des hormones corticosurrénaliennes (glucocorticoïdes, minéralocorticoïdes et androgènes). Le plus souvent, la symptomatologie est d’installation insidieuse, et le caractère non spécifique des symptômes peut être à l’origine d’un retard diagnostic. C’est ainsi qu’il peut n’être porté qu’à l’occasion d’une décompensation aigue, complication majeur engageant le pronostic vital. Bien que rarement décrite dans la littérature [2]; la maladie d’Addison peut se présenter comme un tableau d’insuffisance rénale aigue. Notre cas discutera de la spécificité de cette présentation.

## Patient et observation

**Figure 1:**
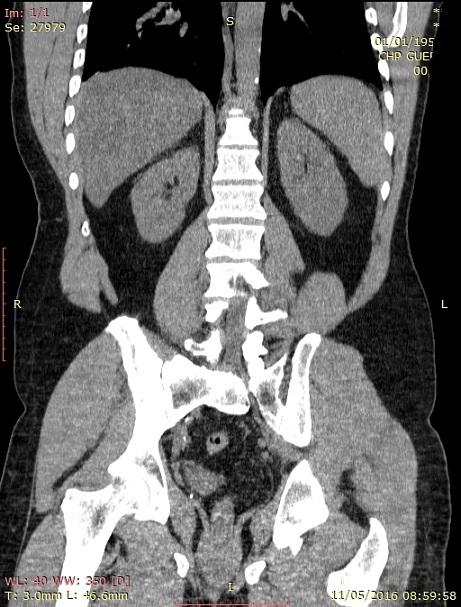
Reconstruction de la loge surrénalienne qui visualise un aspect hypotrophique de la surrénale droite avec une loge surrénale gauche vide

**Figure 2:**
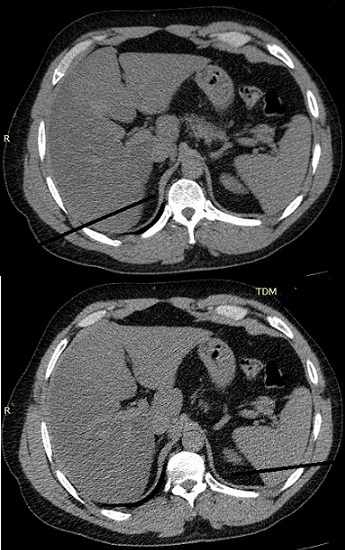
Un aspect hypotrophique de la surrénale droite

Patient de 56 ans, présente depuis 15 jours des nausées, vomissements, asthénie généralisée et douleur osseuse diffuse. Le tout évoluant dans un contexte d’amaigrissement. L’examen clinique trouve un patient altéré, hypotendu à 90/40 mmhg, tachycarde à 100 bpm. Les examens para cliniques objectivent une numération formule sanguine normale, une insuffisance rénale avec urée à 1,85 g/l (0,15-0,45); créatinine 43mg/l (7-14) glycémie à jeun 0,75 g/l (0,7-1,05), hyperkaliémie 7,4 meq/l (3,5-5) et hyponatrémie à 124meq/l (135- 145) ; calcémie corrigée à 110 mg/l (84-96), hyper protidémie à 95mg/l (65-85). Devant l’hyperkaliémie menaçante et la présence de signes électriques à l’ECG; le patient a bénéficié en urgences de deux séances d’hémodialyse; avec une amélioration de la fonction rénale (urée 0,40g/l et créatinine à 14mg/l) et de l’ionogramme sanguin (natrémie 135meq/l et kaliémie à 4,5meq/l). Devant la présence (douleur osseuse+ hyperprotidémie + hypercalcémie); un myélome multiples était suspecté chez ce patient. On a complété par une électrophorèse des protides qui a objectivé une hypo albuminémie sans pic gamma; la protéinurie de 24h00 était négative ; les radiographies standards ainsi que l’échographie rénale étaient normaux. Devant la normalité de ses examens, on a éliminé le diagnostic d’un myélome multiple qui peut être la cause de l’insuffisance rénale aigue chez ce patient. Le patient accusait toujours une asthénie profonde; des nausées. On réexaminant la patient, notre attention a été attirée par la présence d’une hyperpigmentation cutanée, de plaques ardoisées au niveau de la muqueuse buccale. Le diagnostic d’une maladie d’Addison a été suspecté. Un dosage du cortisol était demandé revenant effondré à 1µg/dl confirmant le diagnostic. Le patient était mis sous hydratation par voie intraveineuse et supplémentation par hydrocortisone par voie intraveineuse avec nette amélioration des paramètres cliniques et para cliniques puis relais par hydrocortisone et fludrocortisone par voie orale. Dans le cadre du diagnostic étiologique, TSH est revenue normale à 3,39 µui/ml (0,25-4) avec Anticorps anti TPO négatifs à 0,8 (N < 35). Scanner surrénaliens a objectivé une loge surrénalienne gauche vide avec un aspect hypotrophique de la surrénale droite (Figure 1, Figure 2).

## Discussion

La maladie d’Addison « insuffisance surrénalienne primaire » résulte d’une destruction ou un dysfonctionnement de tout le cortex surrénalien. Bien qu’il s’agisse d’une maladie rare mais son évolution peut être fatale. Son incidence est de 4.7 à 6.2 par million d’habitants [3]. La maladie d’Addison peut se déclarer d’une façon aigue et brutale par une hypotension artérielle, vomissements, diarrhées, douleur abdominale et fièvre [4]. Cependant dans la majorité des cas ; la maladie d’Addison se manifeste par des signes non spécifiques (asthénie, amaigrissement et des troubles digestives) [5]. La mélanodermie est pathognomonique et témoigne de l’origine primitive de l’insuffisance surrénalienne. En dehors de tout contexte d’urgence, le diagnostic positif repose sur le dosage de la cortisolémie à 8 heures du matin est l’examen à réaliser en 1re intention en cas de suspicion d’insuffisance surrénalienne [6]. Le dosage du cortisol chez notre patient est revenu effondrer à 1µg/dl ce qui a confirmé le diagnostic. Un taux d’ACTH élevée permet de confirmer son origine primaire. Pour le diagnostic étiologiques un dosage des AC anti surrénalien et une TDM surrénaliennes sont requis [7, 8]. Bien que rarement rapporté, la maladie d’Addison est une cause d’insuffisance rénale aigue [9]. En effet; les minéralocorticoïdes favorisent la réabsorption de sodium en échange de la sécrétion de potassium et d’hydrogène au niveau du rein ; chose qui explique la survenue de l’hyponatrémie et de l’hyperkaliémie en cas de déficit en minéralocorticoïdes. Ces derniers augmentent aussi le tonus vasculaire et l’inotropisme cardiaque. Par contre ; les glucocorticoïdes augmentent la filtration glomérulaire rénale et l’excrétion de sodium dans les urines mais aussi ils potentialisent l’effet vasoconstricteur des catécholamines sur le plan cardiovasculaire [2]. La combinaison du déficit en glucocorticoïdes et des minéralocorticoides induit une réduction du volume extracellulaire et une baisse du débit cardiaque avec comme conséquence une réduction du débit de perfusion rénale ainsi que du débit de filtration glomérulaire. Ce fut le mécanisme le plus probable expliquant la survenue de l’insuffisance rénale aigue chez ce patient.

A partir de ce cas clinique; on doit discuter un certains nombres de points: la non spécificité des signes cliniques de la maladie d’Addison peuvent être responsable d’un retard diagnostic et de prise en charge thérapeutique; L’association d’une augmentation de l’urée; l’hyperkaliémie et l’hyponatrémie dans la maladie d’Addison peuvent être considérer comme une manifestation de l’insuffisance rénale aigue. La présence d’une hypercalcémie (diminution de l´élimination rénale et augmentation de la réabsorption tubulaire du calcium suivant les mouvements du sodium [10, 11] et d’une hyper protidémie qui sont constaté aussi dans la maladie d’Addison a induit en erreur le clinicien d’évoquer le diagnostic d’un myélome multiple comme cause de l’insuffisance rénale chez ce patient. L’hyperkaliémie dans la maladie d’Addison peut être potentiellement dangereuse. le traitement usuel de l’hyperkaliémie par insuline par voie intraveineuse et de glucose doit être proscrit dans ce cas; car c’est inutile et dangereux chez ses patients prédisposés à faire des hypoglycémies à cause de l’hypocorticisme. L’hyperkaliémie régresse dés mise sous hydratation par sérum salé et hydrocortisone par voise intraveineuse. Dans notre cas fort heureusement; le patient a bénéficié de deux séances d’hémodialyse pour traiter l’hyperkaliémie grave.

## Conclusion

Bien que rarement rapportée; la maladie d’Addison est une cause de l’insuffisance rénale aigue dont l’évolution peut être fatale en l’absence d’un diagnostic précoce et une prise en charge initialement correcte basée sur la réhydratation par sérum salé et hydrocortisone par voie intraveineuse qui permettent une amélioration spectaculaire de la symptomatologie.
